# Mapping few-femtosecond slices of ultra-relativistic electron bunches

**DOI:** 10.1038/s41598-017-02184-3

**Published:** 2017-05-25

**Authors:** Tim Plath, Christoph Lechner, Velizar Miltchev, Philipp Amstutz, Nagitha Ekanayake, Leslie Lamberto Lazzarino, Theophilos Maltezopoulos, Jörn Bödewadt, Tim Laarmann, Jörg Roßbach

**Affiliations:** 1 Department of Physics, University of Hamburg, Luruper Chaussee 149, 22761 Hamburg, Germany; 2 Deutsches Elektronen-Synchrotron DESY, Notkestrasse 85, 22607 Hamburg, Germany; 3 Department of Chemistry, Michigan State University, 578 South Shaw Lane, East Lansing, MI, 48824 USA; 4 European XFEL GmbH, Holzkoppel 4, 22869 Schenefeld, Germany

## Abstract

Free-electron lasers are unique sources of intense and ultra-short x-ray pulses that led to major scientific breakthroughs across disciplines from matter to materials and life sciences. The essential element of these devices are micrometer-sized electron bunches with high peak currents, low energy spread, and low emittance. Advanced FEL concepts such as seeded amplifiers rely on the capability of analyzing and controlling the electron beam properties with few-femtosecond time resolution. One major challenge is to extract tomographic slice parameters instead of projected electron beam properties. Here, we demonstrate that a radio-frequency deflector in combination with a dipole spectrometer not only allows for single-shot extraction of a seeded FEL pulse profile, but also provides information on the electron slice emittance and energy spread. The seeded FEL power profile can be directly related to the derived slice emittance as a function of intra-bunch coordinate with a resolution down to a few femtoseconds.

## Introduction

Pushing the temporal and spectral qualities of high-brightness electron beams to their limits and beyond boosts the performance of free-electron lasers (FELs) since their invention^1–4^, having a transformative impact on science with x-rays^5–9^. In their seminal theoretical work on high-gain x-ray FELs in 1982, Derbenev, Kondratenko and Saldin concluded that the creation of such devices is within the scope of present-day accelerator technology^4^. However, it still took 25 years from the first theoretical considerations to the experimental realization. In 2007, the free-electron laser FLASH at DESY was operational in the so-called water window^10^, where biological function of macromolecules can be imaged in natural environment. Since then, great progress in the development and operation of FELs for the production of ultra-intense soft and hard x-ray pulses down to the Angstrom level has been made^11–13^. The success of FEL science and technology is closely linked to major advances in electron and photon beam diagnostics. The development of novel methods and tools allow for analysis and control of x-ray beam properties with unprecedented femtosecond (fs) temporal precision^14–16^.

Compared to conventional laser technology, FELs have the advantage that important properties of the emitted radiation can be derived by characterizing the FEL gain medium, i.e. the ultra-relativistic electron bunch in the accelerator. In a non-destructive way, it provides few-fs time-resolved information on x-ray pulse properties for each FEL shot and is necessary for all kinds of photon experiments which are sensitive to the photon beam intensity^17^. A prominent example is the nonlinear light-matter interaction in the limit of short wavelength pioneered by Wabnitz *et al*.^18^. Here, the precise knowledge of the light wave characteristics on the fs timescale is a prerequisite for major breakthroughs in FEL science^19^. In nanoscopic systems, the soft X-ray flash deposits a large number of photons per atom within femtoseconds driving extreme states of matter far from equilibrium^20, 21^. Thus, transient dynamics of solid-density material at a resulting temperature of 10^5^–10^6^ K can be studied in the laboratory, which is of great interest in high-pressure and astrophysics, especially planetary science and inertial confinement fusion. Online information on the temporal x-ray pulse profile is particularly crucial for unravelling details of the strong-field interaction in experiments making use of self-amplified spontaneous emission (SASE) FEL pulses. In this mode of operation the exponential gain process of the FEL is started from spontaneous undulator radiation (shot noise)^3^. Thus, the temporal and spectral pulse shape fluctuates significantly from shot to shot. In the so-called data-tagging concept simultaneously recorded experimental data is sorted and evaluated according to the relevant photon beam parameters and observables for each FEL pulse^22^.

The ability of a posteriori optimization of the scientific outcome by correlating the excitation pulse properties with the light-induced sample response on a single-shot basis is only one aspect of successful FEL experimental campaigns. Of course, key accelerator parameters for reliable FEL performance need to be kept under control during its operation, such as energy spread and emittance of the electron beam. Sophisticated timing and feedback systems that steer the individual electron bunch trajectories require detailed information on electron energy and density as function of the intra-bunch coordinate. In particular for generating the shortest photon pulses of a few-fs length or even shorter, the operation of accelerators with low electron bunch charges is challenging^23, 24^. Ultimate control of electron bunch slice properties lead to novel FEL operation schemes such as fresh-slice multicolor lasing with variable polarization of the x-rays^25^.

Recently, temporal coherence provided by so-called seeded FELs moved into the focus of interest^12, 26, 27^. These modern soft x-ray FEL concepts based on the interaction of relativistic electron bunches with external laser pulses give the opportunity to tailor the time-frequency spectrum of the photon beams on demand^28^. Among them, the high-gain harmonic generation (HGHG) FEL has proven to be a reliable mode of operation for FEL user facilities^12, 29, 30^. Here, an external light field imprints a sinusoidal modulation to the longitudinal energy distribution of the electron bunch. A subsequent dispersive element translates this energy modulation into a density modulation resulting in a microbunching pattern with the periodicity of the external light field. In such a way, the harmonic content in the electron density distribution allows for coherent emission in an undulator at integer harmonics of the external light field’s fundamental. In contrast to spontaneous emission as a driver for the FEL, the coherent emission of radiation allows to transfer the coherence properties of the external light field to the FEL pulse. The result is a well-defined radiation pulse with a high degree of spatial and temporal coherence. It has been shown that full control over the light phase allows for a new class of light-phase sensitive experiments at short-wavelength FEL facilities^31–33^, such as nonlinear four-wave mixing^34^ and attosecond (1 as = 10^−18^ s) coherent control^35^. These studies demonstrate the close connection between controlling electron beam slice properties and the resulting new opportunities for tailored photon beam applications.

In the present contribution we show a simple, yet elegant derivation of the slice electron emittance from monitoring the longitudinal distribution using a radiofrequency (rf) deflecting structure. As a result, we obtain a detailed map of slice emittance and slice energy spread along the bunch on a single-shot basis. Since laser seeding is a sensitive local probe for the high quality electron beam, we were able to pinpoint the local performance of the HGHG-seeded FEL with fs resolution. The derived seeded FEL power profiles are in agreement with theoretical predictions. Diagnostic tools of this type are of utmost importance for future realization of compact electron guns, electron accelerators, and free-electron laser architectures^36–38^.

## Experiment

The FEL facility FLASH has been delivering high-brightness photon pulses in the soft x-ray and extreme ultra-violet spectral range to the user community since 2005^10^. Both undulator beamlines, FLASH1 and FLASH2, are driven by one super-conducting linear accelerator with electron beam energies up to 1.25 GeV allowing for simultaneous operation with the full 10-Hz macro-pulse repetition rate^39, 40^. At FLASH1, the sFLASH experiment for research and development of external seeding schemes was installed in 2010 upstream of the main undulator.

**Figure 1:**
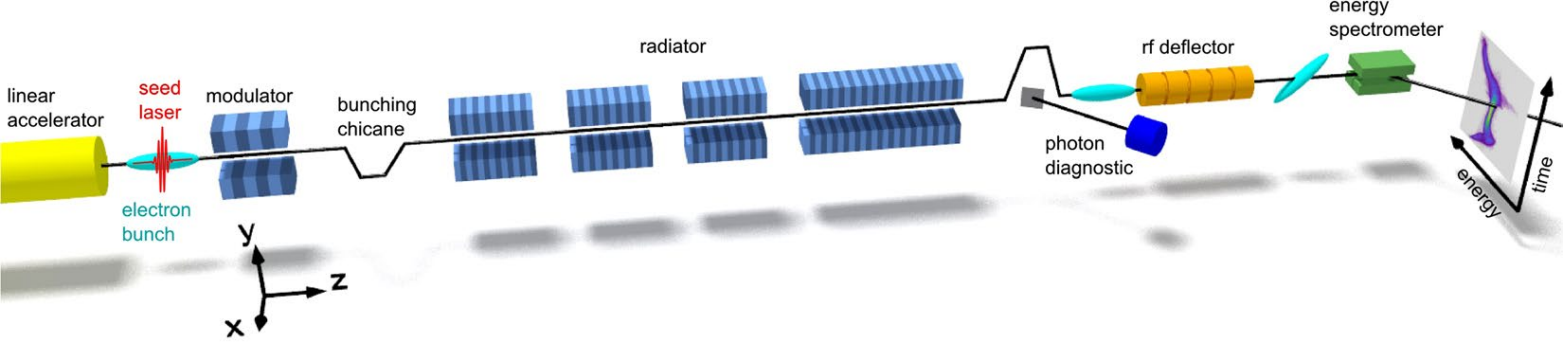
Schematic view of the sFLASH setup. The electron bunch traverses the beamline from left to right coming from the linear accelerator at a kinetic energy of 685 MeV. It is energy modulated at the seed laser wavelength 266 nm in the first long-period undulator. The subsequent chicane transforms the induced energy modulation into a current modulation. In the following undulator modules (radiator) these coherent structures initiate the FEL process at a harmonic of the seed laser wavelength. The electron bunches are characterized with an rf deflector followed by an energy spectrometer, which enables access to the longitudinal phase-space distribution by means of a diagnostic screen (right).

Figure 1 shows a schematic view of the sFLASH setup. It utilizes a 5-period electro-magnetic undulator and a seed laser to imprint a sinusoidal energy modulation onto the electron bunch. The longitudinal dispersion of the subsequent 4-magnetic-dipole chicane transforms this energy modulation into a density modulation with the periodicity of the seed laser. These microbunched electrons coherently emit in the subsequent radiator undulator comprising four modules with peak magnetic fields tuned such that the emission is resonant at the 7th harmonic of the modulation. The key experimental parameters of the present study are given in Table 1.

**Table 1:** Experimental parameters.

Parameter	Value
Electron beam energy	685 MeV
Peak current	0.62 kA
Seed laser wavelength	266 nm
FEL wavelength	38.1 nm
Active undulator length	6.4 m
Chicane dispersive strength *R*_56_	50 μm
Dimensionless Shear parameter of rf-deflector	13.91 ± 0.22

Since the longitudiunal phase-space distribution (LPSD) of the electrons changes significantly when the FEL process reaches saturation, an rf deflector installed at the exit of the radiator is the ideal tool to non-invasively measure the properties of each FEL pulse by characterizing the electron bunch. The rf deflector with a frequency of 2.856 GHz shears the bunch vertically^41, 42^, while a subsequent energy spectrometer deflects the electrons horizontally. Thus, the setup maps the LPSD to the transverse planes which can be observed by a Ce:YAG screen at a temporal resolution of less than 10 fs within the core region of the ultra-relativistic bunch.

**Figure 2:**
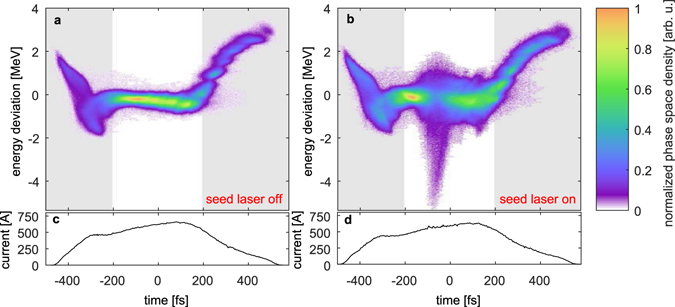
LPSD of an unseeded (**a**) and a seeded electron bunch (**b**) and the corresponding current profiles (**c**) and (**d**). The energy drop and energy spread increase of the electrons due to the FEL process can be seen in the right picture. In the subsequent plots, only the core region (white background) between −200 fs and +200 fs that supports FEL lasing will be shown.

Figure 2 shows a typical measurement of an unseeded and a seeded electron bunch in panels a and b, and the respective current profiles in c and d. The reference of the time axis is the center-of-mass of the charge distribution within the electron bunch. Due to FEL action, the seeded portion loses energy with respect to the unmodulated reference bunch and exhibits a slice energy spread increase, which is clearly visible in Fig. 2b. The drop of the mean energy can be used to directly reconstruct the FEL pulse power profile^17, 43^. The local FEL pulse power *P*(*t*_*i*_) is directly related to the change of the average slice energy ${\rm{\Delta }}E({t}_{i})={E}_{r}({t}_{i})-{E}_{s}({t}_{i})$:

1 $$P({t}_{i})={\rm{\Delta }}E({t}_{i})I({t}_{i})/e,$$

where *I*(*t*_*i*_) is the current at the intra-bunch time *t*_*i*_, *E*_*r*_(*t*_*i*_) is the slice energy of an unseeded reference bunch, *E*_*s*_(*t*_*i*_) the slice energy of seeded bunch, and *e* is the elementary charge.

The reference energy *E*_*r*_ is given by the mean energy of an unseeded electron bunches which only undergoes an FEL process starting from shot noise and does not reach saturation. The corresponding extracted energy within the effective undulator length is about 2–3 orders of magnitude below the energy extracted from the seeded FEL pulses and thus, the impact of self-amplified spontaneous emission on the LPSD is negligible.

**Figure 3:**
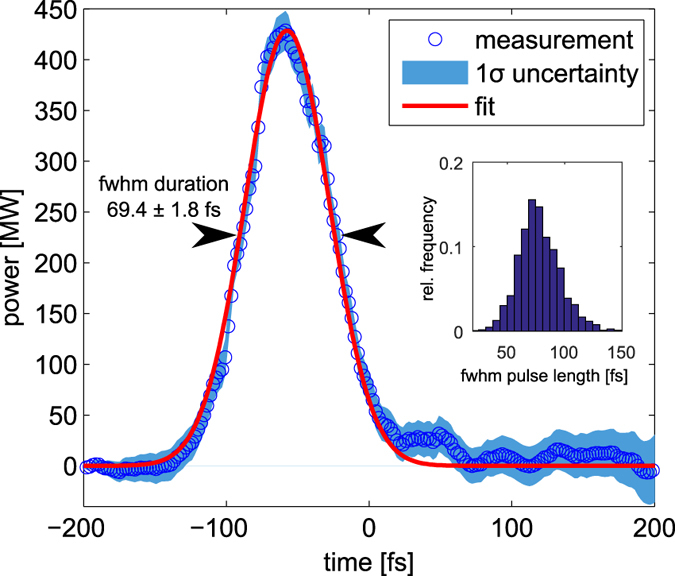
Reconstructed power profile from a typical seeded FEL pulse shown in Fig. 2b using the energy drop method. The blue dots show the reconstruction and the red curve shows a Gaussian fit with a peak power of *P*_0_ = 429 MW. The shaded blue area shows the rms variation of the data derived from statistical errors of the reference bunches. The histogram inset shows the pulse length evaluation of 1979 shots of the present experimental campaign leading to *τ*_*FEL*_ = (78.0 ± 18.4) fs.

A typical FEL power profile reconstructed from a single-shot LPSD measurement is shown in Fig. 3. The blue data points are the reconstruction of the FEL profile according to Eq. 1 and the blue shaded area is the 1 *σ*-uncertainty of the data derived from statistical errors of the reference bunches. For low signal strength at the head and the tail of the pulse the reconstruction algorithm is sensitive to instabilities of the accelerating rf and affected by proper image processing. Thus, a Gaussian function is fitted to the data points for robust data analysis. The fit parameters are the rms pulse duration and central peak position. The optimum fit function is plotted as a red line in Fig. 3. For the purpose of the following analysis, photon pulses with peak powers below 100 MW were disregarded, since these shots have a too low signal-to-noise ratio.

**Figure 4:**
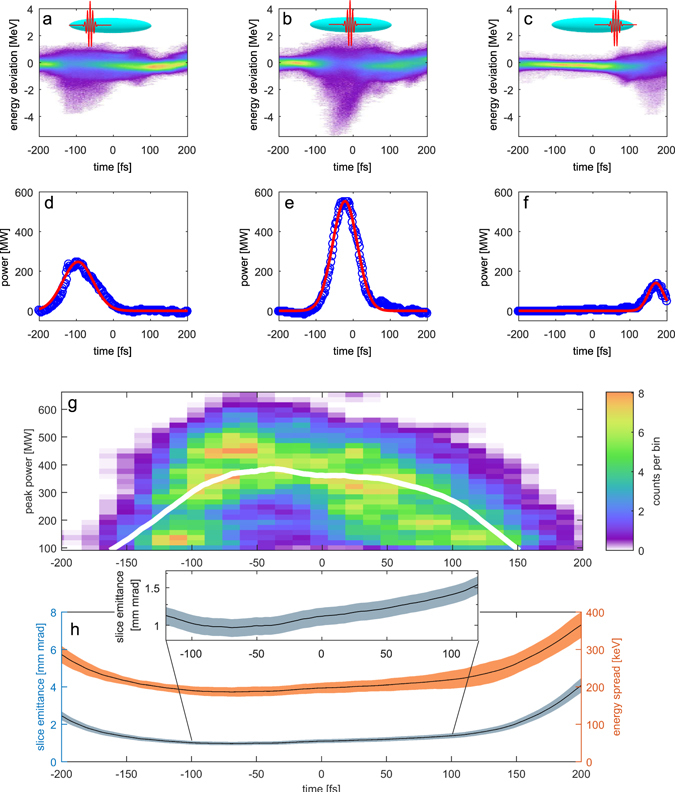
The longitudinal phase space distribution of ultra-relativistic electron bunches for three different relative timings (−97 fs, −21 fs and 168 fs) between seed pulse and electron bunch are shown in panels (**a**–**c**). The panels (**d**–**f**) display the corresponding reconstructed power profiles. The two-dimensional histogram of panel *g* shows the correlation between seeded FEL peak power and the relative position of the seed laser pulse maximum in time. The histogram uses Gaussian smoothing with an rms size of 1 pixel. The white line indicates the seeded FEL performance predicted with the semi-analytical Ming-Xie formalism using electron beam parameters extracted with no FEL action at all. Panel h shows the measured energy spread from the unmodulated reference bunches and the reconstructed emittance used to calculate the prediction in panel g. The inset shows the longitudinal profile of the slice emittance in the core region of the bunch. It has its minimum at about −50 fs and increases towards the outer wings of the scan in agreement with the measured reduction of the emitted FEL power in these regions.

To determine which slices of the electron bunch support high FEL performance, the relative timing between seed laser and electron bunch was varied. With the rf-deflector method described above, the FEL pulse peak power and the longitudinal position of the laser pulse with respect to the charge center of mass can be measured. Typical LPSDs and the corresponding power profiles for different laser-electron timing are shown in Fig. 4a–c and d–f, respectively. Figure 4g correlates the central position of the seed laser and the resulting peak power of 1979 seeded FEL pulses. Fluctuations of the seed pulse intensity and its pointing cause the initital electron energy modulation and thus electron bunching to be different from shot to shot. The jitter of the FEL peak power within the high-power region (−100 fs to +50 fs) of the electron bunch is about 30%. The average seeded FEL peak power obtained within this region has been measured to be 409 ± 110 MW and the average pulse duration is 78.0 ± 18.4 fs as depicted in the histogram in Fig. 3. The average pulse energy is 35.1 ± 10.4 μJ depending on the relative timing between seed pulse and electron bunch in the high power region.

To explain the measured correlation shown in Fig. 4g, the impact of the electron bunch slice parameters on the FEL performance has to be considered. The FEL pulse gains power exponentially, while traversing the undulator magnets in overlap with the electron bunch. The length over which the power grows by a factor of *e* is the gain length *L*_*g*_, which is given by ${L}_{g0}={\lambda }_{U}/(4\pi \sqrt{3}\rho )$ in the one-dimensional theory. Here, $\rho (I,{\sigma }_{{\rm{E}}},{\epsilon }_{n})$ is the Pierce parameter, *λ*_*U*_ is the undulator period, *σ*_E_ is the slice energy spread, and *ε*_*n*_ is the normalized slice emittance. Using the semi-analytical Ming-Xie formalism^44^ that estimates the FEL performance from these one-dimensional parameters, we can now estimate the gain length *L*_*g*_ and the saturation power ${P}_{sat}=1.6\,\rho {P}_{beam}$ of each slice within the electron beam as a function of *I*, *σ*_*E*_, and *ε*_*n*_. Here, *σ*_*E*_ is the slice energy spread that is a superposition of the modulation amplitude Δ*γ* induced by the seed laser and the initial energy spread of the electron beam *σ*_*E*,0_. The latter is known to be a linear function of the peak current *σ*_*E*,0_ ≈ 100/ · *I*_peak_^45^ within the core region of the electron bunch. This function estimates the energy spread caused by the compression of the electron bunch and was corroborated by numerical simulation using the particle tracking codes Astra^46^ and CSRTrack^47^. With the knowledge of the bunch charge, the current can directly be obtained from the measurement of the LPSD of the electron bunch. Finally, the slice emittance has to be reconstructed from the measured energy spread *σ*_*E*,*m*_ of an unmodulated electron bunch as described in the following paragraph.

For extraction of the slice emittance, the different contributions to the observed energy spread *σ*_*E*,*m*_ have to be investigated. Besides the initial slice energy spread *σ*_*E*,0_ of the unmodulated electron beam, there are two more contributions added by the experimental apparatus to the measured slice energy spread *σ*_*E*,*m*_: The deflecting rf-fields in the cavity induce the slice energy spread ${\sigma }_{PW}=K\cdot {E}_{0}\cdot \sqrt{{\beta }_{y}{\epsilon }_{n,y}/\gamma }$, an effect which is along the lines of the Panofsky-Wenzel theorem^48, 49^. *K* denotes the kick parameter of the rf deflector, *β*_*y*_ is the vertical beta-function, *ε*_*n*,*y*_ the vertical normalized emittance, *γ* is the relativistic Lorentz-factor, and *E*_0_ = *γm*_0_ *c* ^2^ the energy of the electron beam. Additionally, the horizontal size of the slice at the observation screen contributes with ${\sigma }_{geom}=A\cdot {E}_{0}\cdot \sqrt{{\beta }_{x}{\epsilon }_{n,x}/\gamma }$, where *A* is the geometrical calibration constant of the screen, *β*_*x*_ and *ε*_*n*,*x*_ are beta-function and normalized emittance in the horizontal plane, respectively. Thus, the total measured energy spread accounts to^50^

2 $${\sigma }_{E,m}^{2}={\sigma }_{E\mathrm{,0}}^{2}+{\sigma }_{PW}^{2}+{\sigma }_{geom}^{2}\mathrm{.}$$

The contributions from the horizontal beamsize and the Panofsky-Wenzel effect are a function of the slice emittance and enable its extraction from the LPSD measurements of unmodulated reference bunches, assuming *ε*_*n*,*x*_ ≈ *ε*_*n*,*y*_ = *ε*_*n*_, (see Supplementary Note 1). These two contributions then account to

3 $${\sigma }_{PW}^{2}(t)+{\sigma }_{geom}^{2}(t)=({K}^{2}{\beta }_{y}+{A}^{2}{\beta }_{x})\gamma {({m}_{0}{c}^{2})}^{2}{\varepsilon }_{n}(t)=\xi {\epsilon }_{n}(t).$$

Inserting this expression into Eq. 2 gives an expression for the slice emittance *ε*_*n*_(*t*):

4 $${\epsilon }_{n}(t)=\frac{{\sigma }_{E,m}^{2}(t)-{\sigma }_{E,0}^{2}(t)}{\xi }.$$

As defined in Eq. 3, *ξ* can be calculated with the knowledge of the measured shear parameter of the rf deflector, the optics, and the calibrations constant of the screen.

In Fig. 4h the average measured slice energy spread 〈*σ*_*E*,*m*_〉 of the unmodulated electron bunches and its standard deviation $\sqrt{{\rm{Var}}({\sigma }_{E,m})}$ from *N*_*r*_ = 189 reference bunches, as well as the slice emittance determined using the method described above together with the statistical error derived from the errors of *σ*_*E*,*m*_ are shown as a function of the intra-bunch coordinate. The projection of the calculated slice emittance along the bunch can be determined with the knowledge of the current profile and is given by *ε*_*n*,proj_ = (3.0 ± 0.3) mm mrad, which is well in line with the projected emittance *ε*_n,match_ = (3.4 ± 0.2) mm mrad obtained during the matching procedure. For a comprehensive analysis of systematic and statistical errors, as well as an in-depth discussion on the implications of the initial model assumptions, see the Supplementary Note.

The white line in Fig. 4g shows the estimated seeded FEL peak power after 6.4 m of effective undulator length according to the model. The only free parameter of the model is the initial modulation amplitude induced by the seed laser and thus the initial bunching that initiates the lasing process. The model’s prediction was fitted to the center-of-mass of each timing bin of the correlation. The best fit gives an initial modulation amplitude of Δ*γ* = 0.777 ± 0.001_stat_ ± 0.154_sys_, well in line with the independent measurement of an uncompressed bunch showing Δ*γ* = 0.79 ± 0.01 for the modulation amplitude. The bunching with a dispersive strength of 50 μm is thus *b* = (3.22 ± 0.03_stat_ ± 1.70_sys_) · 10^−2^. The fit was conducted over the central region of the electron bunch (−125 fs to +125 fs).

## Summary and Outlook

A radio-frequency deflector downstream of the radiator of an FEL is the ideal tool to non-destructively measure the longitudinal power profile of the emitted laser light generated from the signature imprinted to the electron bunches. Furthermore, important electron beam parameters, i.e. slice energy spread and slice emittance are derived by evaluating the measured phase space distribution of unseeded electron bunches upon lasing. With these slice parameters at hand we were able to predict quantitatively the performance of the seeded FEL configuration with an established semi-analytical model. The recorded FEL peak power as a function of the relative timing between the optical femtosecond laser seed and the electron bunch is well described in our model. The required energy modulation amplitude imprinted by the optical laser is in good agreement with measurements of the uncompressed bunch.

The presented study allows to map the regions of the electron bunch, that are capable of lasing with femtosecond resolution. The required assumptions on the initial energy spread restrict its applications to soft x-ray facilities, where collective effects do not spoil the bunch properties significantly. Experimental access to slice parameters of the electron bunch helps to tune the machine for optimum performance. Above all, the effect of inhomogeneities along the electron bunch on the interaction with optical seed pulses become visible and can be studied in great detail on the micrometer length and femtosecond time scale, respectively. On the one hand, this may lead to new strategies avoiding electron beam instabilities and collective effects that hamper reliable seeded operation of these powerful machines for sophisticated user experiments. On the other hand, novel phase-coherent multicolor lasing schemes - possibly with low bunch charges in order to reach the shortest pulses - will rely on the presented capability to analyse and control ultra relativistic electron and photon beams on their intrinsic timescale, which is essentially given by the speed of light.

## Methods

### Seeding setup

The seed laser pulse is generated by frequency tripling of 800 nm laser pulses using a pair of beta barium borate crystals, an intermediate dual-wavelength wave plate, and a alpha barium borate plate to adjust the time delay between the fundamental and the second harmonic radiation. The resulting ultra-violet pulses at 266 nm have a pulse duration of 200 fs (FWHM) and a pulse energy of ≈125 μJ at the interaction point with the electron beam. The relative laser-electron timing is controlled by delaying the optical reference signal fed into the all-optical synchronization of the seed laser oscillator. This setup enables a shot-to-shot stability of the oscillator synchronization of 20 fs rms and an laser-electron synchronization stability of 45 fs rms.

The seeding experiment at FLASH facility utilizes a 5-period electro-magnetic undulator with a period of 20 cm and a maximum undulator parameter of ${K}_{{\rm{mod}}}^{{\rm{\max }}}=10.8$, a subsequent magnetic chicane to generate the necessary longitudinal dispersion of up to 200 µm, and a variable-gap undulator system consisting of three 2-m-long modules with a period of 3.14 cm and one 4-m-long module with a period of 3.3 cm giving a total undulator length of 10 m. Measurements of the gain curve for several different seed laser energies show that the active undulator length is about 6.4 m. The peak values of the undulator K-parameters are ${K}_{\mathrm{rad},1-{\rm{3}}}^{{\rm{\max }}}=2.72$ and ${K}_{\mathrm{rad},4}^{{\rm{\max }}}=3.03$. A magnetic chicane after the undulator guides the electron beam around a mirror used to extract the FEL radiation from the accelerator beam axis. The electron beam afterwards travels through an radio-frequency deflector and an energy spectrometer with the subsequent beam dump.

### Radio-frequency deflector setup

The deflector setup employs a cavity fed with 2.856 GHz radio-frequency to introduce a correlation between electron arrival time and vertical kick. The virtual voltage used during the measurements was 11.7 ± 0.2 MV. The subsequent dipole induces a dispersion of 880 mm and deflects the electron beam onto a Ce:YAG scintillator screen (40 mm × 30 mm, 100 μm thickness) mounted at 45° with respect to the beam axis that is used to measure the longitudinal phase space distribution. This screen is imaged by a camera system mounted perpendicular to the beam axis. The charge-coupled device (CCD) chip of the camera has 1360 × 1024 pixels with 6.4 × 6.4 μm^2^ area per pixel and 12 bit depth at 10 Hz repetition rate. The optical system imaging the screen to the camera leads to an apparent pixel size of 15.4 × 15.4 μm^2^. The post-processing of the CCD images made use of standard image processing techniques.

### Error analysis on emittance

The uncertainties of the emittance shown in Fig. 4g are only derived from statistical errors of the observed energy spread *σ*_E,m_. There are, however, more contributions to possible uncertainties of the emittance than this. Systematic deviations are introduced by lifting the restriction ε_*n*,*x*_ ≈ ε_*n*,*y*_ = ε_*n*_ or a mismatch along the electron bunch caused by collective effects. These effects increase the total relative uncertainty of the emittance to 30%. More information can be found in Supplementary Note 1.

### Fitting Procedure

The free parameter of the semi-analytical model presented is the initial modulation amplitude and bunching. It does not only show a statistical error derived from the fit procedure, but also systematic errors. This systematic uncertainty is induced by the uncertainties of the emittance extraction. A lower emittance would require a lower modulation amplitude and bunching for the same prediction. These errors are estimated by error propagation as shown in Supplementary Note 2.

### Measurement of modulation amplitude

The laser-induced energy modulation amplitude is characterized by switching off all chicanes and using uncompressed electron bunches. That ensures that any effects by longitudinal space charge forces will change the energy distribution along the 20-m beam transport from the modulator undulator to the transverse deflector. The modulation amplitude is visible as an increased local energy spread. Due to the reduced charge density inside the modulated portion of the bunch the signal-to-noise level is reduced on the camera. Therefore, the noise cutting algorithm might cut part of the signal which leads to an underestimation of the modulation amplitude.

## Electronic supplementary material

Supplementary Notes
