# Supplementary material for: Mapping few-femtosecond slices of ultra-relativistic electron bunches
Source: Sci Rep. 2017 May 25;7:2431. doi: 10.1038/s41598-017-02184-3 (PMC5445079; doi:10.1038/s41598-017-02184-3)
Supplement: Supplementary file 1 — Supplementary Notes [file 41598_2017_2184_MOESM1_ESM.pdf]

Mapping few-femtosecond slices of ultra-relativistic electron bunches

T. Plath,[1,*] C. Lechner,[2] V. Miltchev,[1] Th. Maltezopoulos,[3,1] Ph. Amstutz,[1] N. Ekanayake,[4] L. L. Lazzarino,[1] J. Bödewadt,[2] T. Laarmann,[2] and J. Roßbach[1]

[1]*Department of Physics, University of Hamburg,*
Luruper Chaussee 149, 22761 Hamburg, Germany

[2]*Deutsches Elektronen-Synchrotron DESY,*
Notkestrasse 85, 22607 Hamburg, Germany

[3]*European XFEL GmbH, Holzkoppel 4, 22869 Schenefeld, Germany*

[4]*Department of Chemistry, Michigan State University,*
578 South Shaw Lane, East Lansing, MI 48824, USA

SUPPLEMENTARY NOTE 1 – UNCERTAINTIES OF EMITTANCE MEASUREMENT

The Ming-Xie formalism used to estimate the FEL performance uses the normalized one-dimensional emittance as a parameter. An upper estimate for this emittance is the geometric mean of the transverse emittances $\epsilon_{\mathrm{mx}}(t_i) = \sqrt{\epsilon_{\mathrm{n,x}}(t_i)\ \epsilon_{\mathrm{n,y}}(t_i)}$ [1]. In the section describing the extraction of the emittance from the energy spread, we initially assumed the transverse emittances are equal. We will start this section by assuming that this is not the case and investigate the uncertainty of $\epsilon_{\mathrm{mx}}(t_i)$ including statistical uncertainties as well as uncertainties arising from violation of our assumptions. For a more convenient notation we will drop the (t_i) from the emittance terms.

If the slice emittances in both planes are not equal, we define the parameter u such that

$$\epsilon_{\mathrm{x,n}} = \epsilon_{\mathrm{n}} \text{ and } \epsilon_{\mathrm{y,n}} = u\,\epsilon_{\mathrm{n}}, \tag{1}$$

and

$$\xi = (K^2\beta_y u + A^2\beta_x)\gamma(m_0c^2)^2. \tag{2}$$

To identify parameters that are affected by uncertainties we have to analyze the full expression for the one-dimensional emittance

$$\epsilon_{\mathrm{mx}}(t_i) = \epsilon_{\mathrm{n}}\sqrt{u} = \frac{\sigma_{\mathrm{E,m}}^2 - \sigma_{\mathrm{E,0}}^2}{(K^2\beta_y u + A^2\beta_x)\gamma(m_0c^2)^2}\ \sqrt{u}. \tag{3}$$

The parameters that are subject to statistical measurement errors are the measured slice energy spread $\sigma_{\mathrm{E,m}}$, the initial energy spread $\sigma_{\mathrm{E,0}}$ (from the current measurement), and the rf deflector kick parameter K. As discussed above, u can deviate from 1, if the transverse emittances are not equal. Additionally, mismatch caused by collective effects can cause β_x and β_y to differ from the optical functions of the machine.

The β-mismatch and emittance deviation u are unknown from the measurement. Start-to-End simulations of similar bunches suggest that the emittance deviation u between both planes is smaller than 10%. The variation of the beta function along the core region of the bunch is in the same order of magnitude. Since we could not measure these quantities Supplementary Fig. 1 shows the relative emittance error as a function of the relative errors in u and the β-functions (same error for both dimensions). If the assumptions are correct, the relative emittance uncertainty is about 17%, and increases with higher values of u and

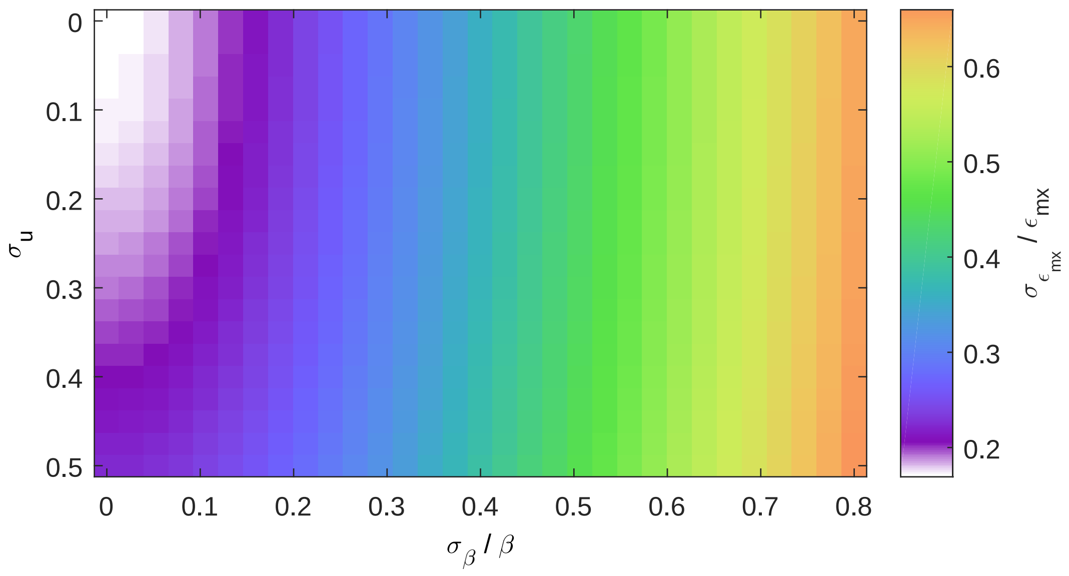

Supplementary Figure 1. Relative emittance error as a function of deviations of u and β-functions from the assumptions.

σ_β/β. The mismatch of the optical functions here has the bigger impact on the error. Since both quantities can only be estimated from numerical simulations, the systematic errors on b_7 use a conservative estimation of $\sigma_{\epsilon_{\rm mx}}/\epsilon_{\rm mx} \approx 0.3$, allowing for $\sigma_\beta/\beta \approx 0.3$.

Since the initial energy spread $\sigma_{\rm E,0}$ is not measured, but only derived from numerical simulations, it can be treated as another error source on the extracted emittance. Assuming an uncertainty of the energy spread of as much as 100% the relative emittance error would only grow to $\sigma_{\epsilon_{\rm mx}}/\epsilon_{\rm mx} \approx 0.36$.

SUPPLEMENTARY NOTE 2 – FITTING THE MODEL TO THE DATA

The power of each slice is calculated from an analytical expression for a seeded gain curve [2]:

$$P(z) = P_{\mathrm{th}} \left[\frac{\frac{1}{3}\left(\frac{z}{L_g}\right)^2}{1+\frac{1}{3}\left(\frac{z}{L_g}\right)^2} + \frac{\frac{1}{2}\exp\left[\frac{z}{L_g}-\sqrt{3}\right]}{1+\frac{P_{\mathrm{th}}}{2P^*_{\mathrm{sat}}}\exp\left[\frac{z}{L_g}-\sqrt{3}\right]} \right], \tag{4}$$

where z is the longitudinal position along the undulator, $P^*_{\mathrm{sat}} = P_{\mathrm{sat}} - P_{\mathrm{th}}$ and $P_{\mathrm{th}} = \rho|b|^2 P_{\mathrm{beam}}$. P_{th} is the power threshold at which the behavior of the power gain switches from the quadratic dependency of coherent radiation to the exponential regime of the free-electron laser.

By varying the initial bunching b this analytical estimation has been fitted to the center-of-mass of each timing bin of Fig. 4g with the means of a χ^2-fit. The uncertainties of the data points are only statistical errors and do not include any systematic errors, e.g. from calibration of the LPSD measurement. The scan shown in Fig. 4g was binned to 31 time bins, the central 21 of which have been used for the fitting procedure. The bins in the wings suffer from poor signal-to-noise ratio. The reduced χ^2 of the fit is about 3.8, indicating a possible underestimation of the error bars.

In addition to the statistical errors derived from the fit, the bunching also shows a systematic error. This error arises from the uncertainty of the emittance. These uncertainties can be treated as systematic error of the model and are propagated to the final fit result:

$$\sigma_{b_7} = \left(\frac{\partial b_7}{\partial \epsilon_{\mathrm{mx}}}\right) \sigma_{\epsilon_{\mathrm{mx}}}. \tag{5}$$

---

* tim.plath@desy.de

[1] E. Prat and M. Aiba, Phys. Rev. ST Accel. Beams **17**, 052801 (2014).

[2] L. Gianessi, *Seeding and Harmonic Generation in Free-Electron Lasers* in E. Jaeschke, S. Khan, J. R. Schneider, and J. B. Hastings (Eds.), *Synchrotron Light Sources and Free-Electron Lasers* (Springer International Publishing, 2016).
